# The Rate of *Helicobacter pylori* Seropositivity in a Group of Korean Patients with HLA-B27-Associated Acute Anterior Uveitis

**DOI:** 10.1371/journal.pone.0123924

**Published:** 2015-04-20

**Authors:** Jeong Hun Bae, Joon Mo Kim

**Affiliations:** Department of Ophthalmology, Kangbuk Samsung Hospital, Sungkyunkwan University School of Medicine, Seoul, Republic of Korea; Oregon Health & Science University, UNITED STATES

## Abstract

**Purpose:**

To investigate an association between *Helicobacter pylori* seropositivity and HLA-B27-positive acute anterior uveitis (AAU) in Korean patients.

**Methods:**

Retrospective analysis was performed with data from 106 patients previously diagnosed with AAU without clinical evidence of spondyloarthropathy. Serum immunoglobulin G antibodies to *H*. *pylori* were measured by enzyme-linked immunosorbent assay, and HLA typing was performed using polymerase chain reaction of DNA amplification. We included 72 non-uveitis patients and 35 age- and sex-matched healthy controls in the study.

**Results:**

Of the 106 patients with AAU, 41 (38.7%) were HLA-B27-positive, and 45 (42.5%) were seropositive for *H*. *pylori*. Patients with HLA-B27-positive AAU had a significantly lower prevalence of *H*. *pylori* seropositivity compared to those with HLA-B27-negative AAU and healthy controls (24.4% *vs*. 53.8%, *p* = 0.003; 24.4% *vs*. 57.1%, *p* = 0.004, respectively). In the non-uveitis group, however, HLA-B27-positive patients exhibited similar *H*. *pylori* seropositivity prevalence to HLA-B27-negative patients and healthy controls (45.5% *vs*. 55.7%, *p* = 0.529; 45.5% *vs*. 57.1%, *p* = 0.497, respectively). In multivariate analysis, a low prevalence of *H*. *pylori* seropositivity was significantly associated with HLA-B27-positive AAU (odds ratio = 0.340, 95% confidence interval 0.135–0.855, *p* = 0.022).

**Conclusions:**

Our results suggest an inverse association between *H*. *pylori* seropositivity and HLA-B27-positive AAU. Further investigation of this association is needed, given the low prevalence of *H*. *pylori* seropositivity observed in patients with HLA-B27-positive AAU.

## Introduction

Acute anterior uveitis (AAU) is a common form of intraocular inflammation, with more than 50% of patients with AAU reported to be HLA-B27-positive in a Caucasian population [1]. HLA-B27-positive uveitis has been associated with seronegative spondyloarthropathies such as ankylosing spondylitis, reactive arthritis, and psoriatic arthropathy. However, the pathogenesis of HLA-B27-associated diseases is not clearly understood, and likely involves both genetic and environmental factors [2]. Gram-negative bacteria, such as *Klebsiella*, *Salmonella*, *Yersinia*, and *Chlamydia* species, have been implicated in the pathophysiology of HLA-B27-positive AAU and several spondyloarthropathies [3–6].

*Helicobacter pylori* is a gram-negative bacterium that causes one of the most common infections in humans. The prevalence of *H*. *pylori* seropositivity increases with age, affecting more than 80% of middle-aged adults in developing countries and 20–50% in developed countries [7]. In Korea, the overall prevalence was estimated at 56.0% in 15,916 healthy subjects aged 16 years or older [8]. Despite the high prevalence of *H*. *pylori*, only a small group of infected patients develop gastrointestinal symptoms, likely due to different levels of virulence and the nature of the host immune response [9]. *H*. *pylori*, like other gram-negative bacteria, has lipopolysaccharides that may cause inflammation and thereby stimulate host immunity. *H*. *pylori* has come into the spotlight in ophthalmology because of its possible role in several eye diseases including Sjögren’s syndrome, blepharitis, and central serous chorioretinopathy [10–12]. Anti-*H*. *pylori* antibodies have been detected in the serum of patients with reactive arthritis, Behcet’s disease, and glaucoma. Recently, *H*. *pylori* infection has been proposed as an environmental risk factor for uveitis including an HLA-B27-positive subgroup [13]. However, there is no direct evidence that *H*. *pylori* contributes to HLA-B27-positive AAU.

Therefore, we investigated an association between *H*. *pylori* and HLA-B27-positive AAU in Korean patients.

## Materials and Methods

A total of 106 patients diagnosed with AAU without clinical evidence of spondyloarthropathy at the Department of Ophthalmology, Kangbuk Samsung Hospital from January 2011 to June 2013 were included in the study. An ophthalmic examination was performed at each follow-up visit including corrected visual acuity test, intraocular pressure (IOP) measurement, slitlamp biomicroscopy, and a fundus examination with indirect ophthalmoscopy. Ocular inflammation was classified according to the recommendations of the Standardization of Uveitis Nomenclature (SUN) Working Group [14]. Posterior involvement of AAU including retinal vasculitis, papillitis, and cystoid macular edema (CME) was confirmed with fluorescein angiography and optical coherence tomography. Diffuse vitritis was diagnosed based on the presence of 2+ cells or more in the vitreous cavity, in order to differentiate this condition from anterior vitreous spillover cells. Demographic data on age, sex, ocular conditions other than AAU, and systemic diseases were recorded. All patients were referred to the rheumatology department, and a rheumatological evaluation for a history of musculoskeletal, abdominal, and skin diseases, as well as sacroiliac radiological examination were performed. The presence of axial spondyloarthropathy was confirmed by a rheumatologist according to the Assessment of SpondyloArthritis International Society classification criteria [15]. Laboratory tests and IOP measured with a Goldmann applanation tonometer at the initial visit were used for analysis. The medical records of 72 patients without a history of uveitis were reviewed to compare rates of *H*. *pylori* seropositivity. Ocular disorders in the non-uveitis group consisted of normal tension glaucoma, age-related macular degeneration, epiretinal membrane, and retinal vein occlusion. As controls, 35 healthy subjects who visited our clinic for the assessment of age-related cataract were also included. This study was approved by the ethics committee of the Institutional Review Board of Kangbuk Samsung Hospital (IRB No. KBC11141) and conducted according to the tenets of the Declaration of Helsinki. Written informed consent was obtained from all participants.

*H*. *pylori* seropositivity was determined by measuring anti-*H*. *pylori* immunoglobulin G (IgG) antibodies in the serum using enzyme-linked immunosorbent assay (ELISA, Genedia *H*. *pylori* ELISA; Green Cross Medical Science Corp., Seoul, Korea). The level of IgG antibodies to *H*. *pylori* is given in units per milliliter, and antibody levels of > 15 U/ml were considered positive for *H*. *pylori* according to the manufacturer’s recommendation.

The serologic test for HLA-B27 antigen was accomplished by polymerase chain reaction (PCR) of DNA amplification with specific primers of E91s and E136as. Generation of the amplified PCR product was detected on 2% agarose minigel electrophoresis. Positive samples produced a DNA band of 135 bp.

Statistical analysis was performed with the statistical package PASW (Version 18.0, SPSS Inc., Chicago, IL). Mann-Whitney U test and independent samples *t*-test were used for comparison of age and IOP between groups. Associations between HLA-B27 and sex, clinical features, and seropositivity of anti-*H*. *pylori* IgG antibodies were analyzed using Pearson’s chi-square test or Fisher’s exact test. Univariate and multivariate analyses between the prevalence of HLA-B27-positive AAU and clinical variables were performed using a logistic regression model. Odds ratios and 95% confidence intervals are reported. *P* values were two-paired; *p* < 0.05 was considered statistically significant.

## Results

Table 1 summarizes the demographics of participants and the prevalence of *H*. *pylori* seropositivity in each group. Of the 106 patients with AAU, 53 were men and 53 were women, the mean age was 46.0 ± 13.6 years (range, 11–79 years), 41 (38.7%) were HLA-B27 positive. Forty eight patients (45.3%) presented with acute onset of unilateral alternating or bilateral simultaneous anterior uveitis, 57 (53.8%) had had recurrent episodes, and 17 (16.0%) exhibited accompanying posterior involvement. Diffuse vitritis (14 eyes) was the most common feature of posterior involvement, followed by CME (5 eyes) and retinal vasculitis (2 eyes).

**Table 1:** Demographics of participants and the prevalence of *H*. *pylori* seropositivity in the uveitis group, the non-uveitis group, and the control group.

	Uveitis group			Non-uveitis group			Control group
Variable	HLA-B27 (+) (*n* = 41)	HLA-B27 (-) (*n* = 65)	*p* value	HLA-B27 (+) (*n* = 11)	HLA-B27 (-) (*n* = 61)	*p* value	(*n* = 35)
Age (years, mean ± SD)	42.6 ± 14.2	48.1 ± 12.9	0.042^a^ *	45.3 ± 17.4	49.1 ± 14.0	0.531^b^	45.6 ± 15.5
Sex (% of male)	48.8%	50.8%	0.842^c^	54.5%	60.7%	0.747^d^	51.4%
Anti-*H*. *pylori* IgG antibodies (*n*, % of seropositivity)	10 (24.4%)	35 (53.8%)	0.003^c^ *	5 (45.5%)	34 (55.7%)	0.529^c^	20 (57.1%)

IgG: immunoglobulin G; SD: standard deviation.

^a^independent samples t-test

^b^Mann-Whitney U test

^c^Pearson’s chi-square test

^d^Fisher’s exact test.

*p < 0.05.

The prevalence of positive *H*. *pylori* tests was 53.8% in the HLA-B27-negative AAU group, 45.5% in the HLA-B27-positive non-uveitis group, and 55.7% in the HLA-B27-negative non-uveitis group; none of these differed significantly from the prevalence in the control group (57.1%) (*p* = 0.752, *p* = 0.497, *p* = 0.894, respectively). However, patients with HLA-B27-positive AAU had a significantly lower prevalence of positive *H*. *pylori* tests than those with HLA-B27-negative AAU and controls (24.4% *vs*. 53.8%, *p* = 0.003; 24.4% *vs*. 57.1%, *p* = 0.004, respectively, Table 1). A positive titer of IgG antibodies to *H*. *pylori* was inversely associated with HLA-B27-positive AAU (odds ratio = 0.276, 95% confidence interval 0.117–0.656, *p* = 0.003). After adjusting for age, sex, high IOP, and the presence of posterior synechiae, a logistic regression model still showed an inverse association between *H*. *pylori* seropositivity and HLA-B27-positive AAU (odds ratio = 0.340, 95% confidence interval 0.135–0.855, *p* = 0.022, Table 2).

**Table 2:** Logistic regression model for HLA-B27-positive acute anterior uveitis.

	Univariate analysis			Multivariate analysis^†^		
Variable	*p* value	OR	95% CI	*p* value	OR	95% CI
Age	0.046*	0.969	0.940–0.999	0.316	0.984	0.953–1.016
Sex	0.842	1.083	0.495–2.367	0.727	0.854	0.353–2.066
Posterior synechiae	0.056	3.697	1.035–13.201	0.091	3.228	0.830–12.555
Elevated IOP (>25 mmHg)	0.015*	0.110	0.014–0.885	0.062	0.123	0.013–1.115
Seropositive anti-*H*. *pylori* IgG antibodies	0.003*	0.276	0.117–0.656	0.022*	0.340	0.135–0.855

CI: confidence interval; IgG: immunoglobulin G; IOP: intraocular pressure; OR: odds ratio.

*p < 0.05.

^†^Multivariate analysis includes clinically relevant covariates and covariates with *p* < 0.1 on univariate analysis.

In regards to IOP, 12 of the patients with HLA-B27-negative AAU had elevated IOP (> 25 mmHg), while one with HLA-B27-positive AAU had elevated IOP (*p* = 0.015). Eight of the 13 patients with elevated IOP had antibodies to *H*. *pylori*, and the frequency was 17.8% in the *H*. *pylori*-seropositive patients. The difference in mean IOP between HLA-B27-positive AAU and HLA-B27-negative AAU groups was statistically significant (13.3 ± 4.3 mmHg *vs*. 17.1 ± 9.8 mmHg; *p* = 0.008). However, there was no significant difference in mean IOP between *H*. *pylori*-seropositive and *H*. *pylori*-seronegative patients with AAU (17.1 ± 9.3 mmHg *vs*. 14.5 ± 7.4 mmHg; *p* = 0.131). There were no differences in the rate of topical or systemic corticosteroid use between the two groups during the follow-up period.

## Discussion

Our study showed that patients with HLA-B27-positive AAU had a significantly lower prevalence of *H*. *pylori* seropositivity compared to patients with HLA-B27-negative AAU and controls. However, there was no significant difference in the prevalence of *H*. *pylori* seropositivity between the non-uveitis group and the control group. A positive titer of anti-*H*. *pylori* antibodies was significantly associated with low odds ratio for HLA-B27-positive AAU even after adjustment for other confounding factors. These results suggest an inverse relationship between *H*. *pylori* seropositivity and HLA-B27-positive AAU.

The HLA-B27 antigen is considered closely related to AAU, and exogenous peptides from gram-negative bacteria have been suggested to be environmental triggers for HLA-B27-positive AAU [16,17]. Huhtinen et al and Otasevic et al reported the possible role of gram-negative bacteria including *H*. *pylori* in the development and recurrence of HLA-B27-positive AAU [13,18]. On the other hand, Onal et al reported that there was no significant association between gram-negative organisms and AAU [19]. In this study, we showed an inverse association between *H*. *pylori* seropositivity and HLA-B27-positive AAU, given the significantly lower prevalence of *H*. *pylori* seropositivity in patients with HLA-B27-positive AAU than those with HLA-B27-negative AAU and controls. Our results were derived purely from patients with AAU, while other studies included spondyloarthropathies as well as AAU. The role of bacterial infections in HLA-B27-positive spondyloarthropathies has been demonstrated by the presence of some bacterial antigens, DNA, and specific T cells in the synovial fluid [20,21]. However, such direct evidence has not been demonstrated in the uveal tissues of patients with HLA-B27-positive AAU. This suggests that AAU may not share the association of spondyloarthropathies with bacterial infections in HLA-B27-positive patients.

One recent study reported that *H*. *pylori* seropositivity was associated with elevated IOP in anterior uveitis [22]. In our study, *H*. *pylori-*seropositive patients had a higher mean IOP than seronegative patients, though the difference was not statistically significant. Nevertheless, the mean IOP in the HLA-B27-positive AAU group was significantly lower than that in the HLA-B27-negative AAU group, suggesting that HLA-B27-positive AAU is not associated with elevated IOP, and may show different features from AAU in *H*. *pylori*-seropositive patients.

The prevalence of HLA-B27 and *H*. *pylori* seropositivity varies by ethnicity and geography. In Europe, HLA-B27 prevalence was reported as 8–10%; in the United States, the National Health and Nutrition Examination Survey reported the prevalence as 7.5% among non-Hispanic whites and 3.5% among all other ethnicities [17,23]. In Korea, the prevalence of HLA-B27 is 4.8%, which accounts for 35% of patients with AAU [24]. Despite a higher prevalence of *H*. *pylori* seropositivity (56%), however, the prevalence of HLA-B27-positive AAU in Korea is approximately one-third of all AAU cases, while that in western countries is approximately one-half.

As mentioned earlier, there is no clear evidence on an association between HLA-B27-positive AAU and *H*. *pylori* seropositivity. Conflicting results may be due to the varied geographic and ethnic prevalence of HLA-B27 and *H*. *pylori*, genetic polymorphisms, and a host of environmental factors. Furthermore, HLA-B27 has 105 subtypes that differ from each other by exonal polymorphisms, possibly resulting in the diverse uveitogenic and arthritogenic properties of HLA-B27 [25]. It is known that such a polymorphism leads to different associations between HLA-B27 subtypes and diseases such as AAU [26,27].

Given the high prevalence of *H*. *pylori* seropositivity in Korea, our results show an inverse relationship between HLA-B27-positive AAU and *H*. *pylori* seropositivity. In a mouse model, *H*. *pylori* infection was reported to have a protective effect on allergic asthma through the induction of regulatory T cells [28]. *H*. *pylori* would have the immunomodulatory properties mediated by suppressive activity of regulatory T cells. Therefore, the low prevalence of *H*. *pylori* infection might be related to reduced activity of regulatory T cells and consequent development of autoimmune diseases such as HLA-B27-positive AAU. On the other hand, it has been hypothesized that HLA-B27 may modulate gram-negative bacterial invasion into host cells by a specific B27-microbial interaction [29]. The altered response to bacterial infections may also be reflected in the antigen-presenting process of the peripheral blood lymphocytes [30]. This hypothesis was supported by a recent rat study that showed the effects of HLA-B27 on specific alterations in gut microbiota [31]. However, it is unclear from this study whether or not HLA-B27 impairs immune responses to *H*. *pylori* or has a protective role against the organism, and this relationship requires further investigation.

The limitations of our study include its retrospective design and the possibility of selection bias. The small sample size, especially in the HLA-B27-positive non-uveitis group, may undermine the reliability of our results in assessing the prevalence of *H*. *pylori* seropositivity. Furthermore, the socioeconomic status of the participants, which might affect *H*. *pylori* seropositivity, was not evaluated in the study. Future well-designed large cohort studies are warranted to clarify the relationship between *H*. *pylori* and HLA-B27-positive AAU.

In conclusion, our data showed that the positive rate of anti-*H*. *pylori* antibodies was significantly lower in the patients with HLA-B27-positive AAU than those with HLA-B27-negative AAU or controls. After adjusting for confounding factors, the prevalence of *H*. *pylori* seropositivity still remained lower in the HLA-B27-positive AAU patients. Thus, we suggest that *H*. *pylori* seropositivity is inversely associated with HLA-B27-positive AAU.
